# Salt mixtures in stone weathering

**DOI:** 10.1038/s41598-023-40590-y

**Published:** 2023-08-16

**Authors:** Sebastiaan Godts, Scott Allan Orr, Michael Steiger, Amelie Stahlbuhk, Tim De Kock, Julie Desarnaud, Hilde De Clercq, Veerle Cnudde

**Affiliations:** 1 Monuments Lab, Royal Institute for Cultural Heritage (KIK-IRPA), Brussels, Belgium; 2 Antwerp Cultural Heritage Sciences, ARCHES, University of Antwerp, Antwerp, Belgium; 3 Department of Geology, PProGRess, Ghent University, Ghent, Belgium; 4 Institute for Sustainable Heritage, University College London (UCL), London, UK; 5 Department of Chemistry, University of Hamburg, Hamburg, Germany; 6 Laboratoire de Recherche Des Monuments Historiques (LMRH), Paris, France; 7 Department of Earth Sciences, Utrecht University, Utrecht, The Netherlands

**Keywords:** Climate sciences, Environmental sciences, Hydrology, Limnology, Natural hazards, Ocean sciences, Planetary science, Solid Earth sciences, Astronomy and planetary science, Chemistry, Energy science and technology, Engineering, Chemical physics

## Abstract

Salt related weathering of stones has been attributed to pressures exerted by repeated cycles of crystallization within pores. Relative Humidity (RH) is a key driver for dissolution and crystallization processes. Despite the prevalence of salt mixtures in natural environments, most experimental work has focused on single salts. Thus, the identification of salt mixture composition and their behavior is necessary to understand weathering. Thermodynamic calculations are used to analyze several thousand realistic salt mixtures found in weathered stone. We identify two common mixture types and their behavior. From at least 85 salt species theoretically present, 14 common salts are identified that occur most frequently and their critical RH points are discussed. These findings have wide-reaching implications for understanding salt weathering processes and informing the design of experimental stone weathering research.

## Introduction

The behavior of salt mixtures is an important cause of decay and moisture accumulation in porous materials^1,2^. Investigations on the subject have been carried out to understand the chemo-mechanical processes, as detailed by Flatt et al.^3^. However, most studies focus on individual salts^4–11^, overlooking the reality that salts often exist in mixtures and behave differently when compared to single salts ^12–23^. This behavior is the key driver of decay and is caused by crystallization and dissolution cycles triggered by environmental conditions. The relative humidity (RH) or temperature (T) in which critical crystallization can occur is thus determined by the mixture composition.

To illustrate the complexity of realistic salt mixtures and the changes of salt behavior, the following case study is used. The salt content of a decaying limestone in the World Heritage Site, Phare de Cordouan, France is investigated. Instead of just sodium chloride (halite), which has a crystallization RH at approximately 75% (20 °C), a complex mixture of salts was found, similar to the findings described by Prokos^24^. The mixture composition causes halite to crystalize at lower RH, here around 65% (20 °C). This variation influences our understanding of decay patterns and is crucial in assessing the impact of climate on salt crystallization cycles and phase changes.

In theory, more than 85 possible salt phases can form when considering ions that might be found in stones, including relatively common ones (e.g., chloride, nitrate, sulfate, sodium, potassium, magnesium, and calcium), and less common ions (e.g., (bi)carbonate, fluoride, phosphate, nitrite, oxalate, ammonium, acetate, and formate)^25,26^. By limiting focus to common ions, a total of 40 salt phases remain^27^. Commonly found ions have many origins, a typical example can be found in churches where hundreds of buried human remains directly influence the salt type and content deposited in the stone materials over time. Ions from decomposing organic materials, typical ions in groundwater and other contaminations are transported into porous materials by capillary forces, leading to the crystallization of salts at the drying front, followed by the deposit and further creep of more soluble salts at heights beyond the reach of pure water^25,28–31^. Other typical sources of ions originate from the stone materials themselves, interactions with pollutants in the atmosphere, infiltrating rainwater, and products used for maintenance or conservation of building materials^32^.

This research focusses on the identification of common salt mixtures found in the built environment and classifies them into types. The investigation is based on a large dataset of ion measurements for which charge-balanced ion data are used as input for thermodynamic modeling to derive outputs of the mixture crystallization behavior under changing RH. Additionally, the critical crystallization and dissolution ranges of the identified salts are investigated for each mixture composition and compared to the behavior as a single salt.

## Methods

**Figure 1:**
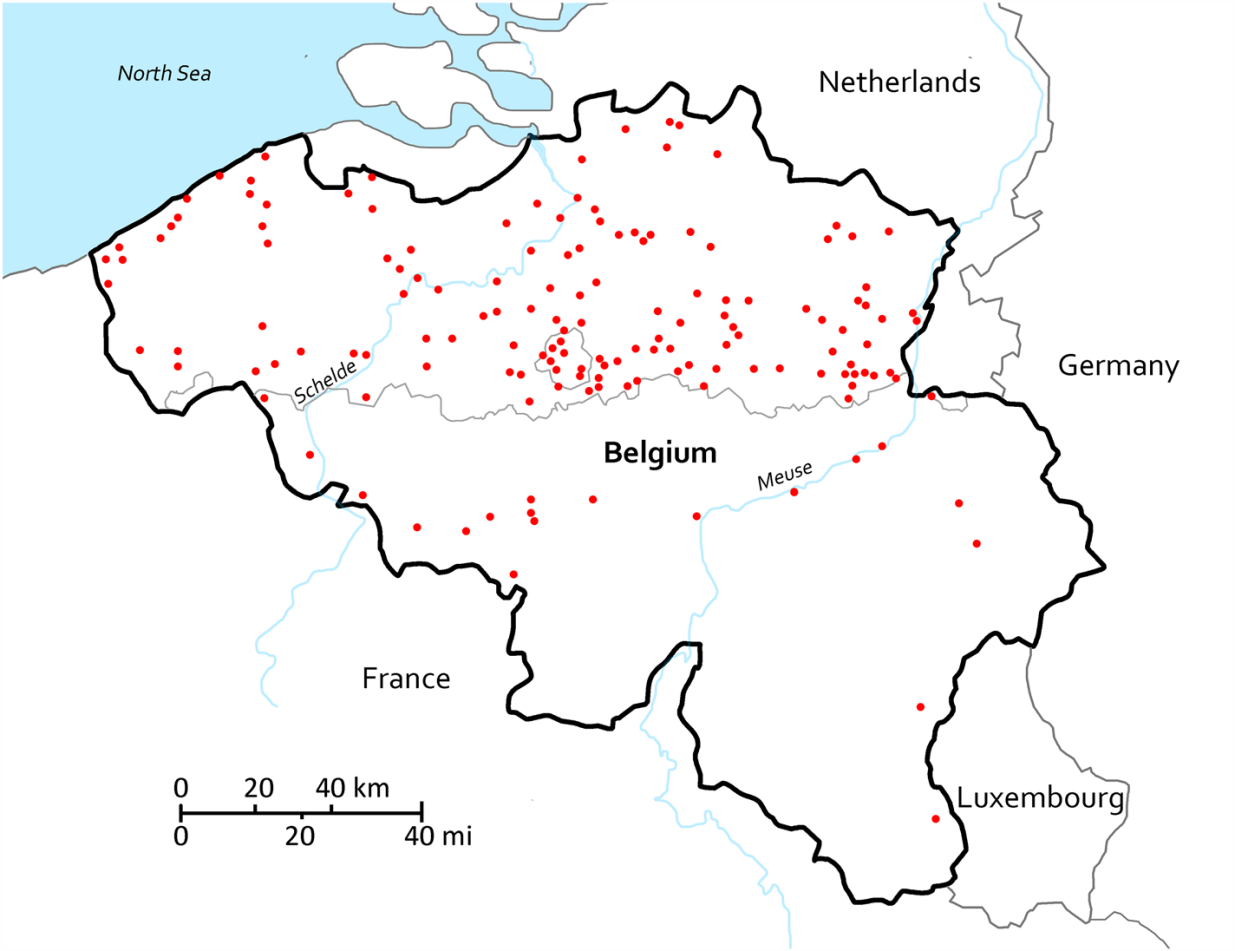
Overview of the locations of 338 sites where in total 11,412 drill samples were taken for the determination of the quantitative ion concentrations of Cl^−^, NO_3_^−^, SO_4_^2−^, Na^+^, K^+^, Mg^2+^, and Ca^2+^. Several sites are located in a single city, for example: 30 in Brussels and 25 in Antwerp. The dataset also includes one site in Germany, one in Italy, two in The Netherlands and four in the Czech Republic. Drawn with Adobe Photoshop 2022, map data available at^35^.

The complete dataset used in this study includes 11,412 drill samples analyzed with ion chromatography, specifically 79,884 ion concentrations of chloride, nitrate, sulfate, sodium, potassium, magnesium, and calcium. The ion analysis and data treatment are extensive and therefore detailed in^33^, while the complete dataset and calculations are available at^34^. The samples are taken from weathered stone materials in 338 monuments, archaeological sites, mural paintings, and sculptures, primarily in Belgium (Fig. 1). The related building materials were mostly produced between the tenth and twentieth centuries and consist of traditional lime-based mortar, cement, plaster (including wall paintings), brick, and natural stone (mainly limestone). The ions detected are to an important degree representative of the diverse mixtures found in stone materials globally.

The identification of common mixtures, solids, and behavior under changing RH conditions is based on the ion dataset, corrected to achieve an equilibrium charge balance within each individual sample. The balanced ion concentrations are presented as mole fraction and equimolar contents of calcium and sulfate, considered as the gypsum content, are removed (further detailed in ^33^), which identified three mixture types:

- Type 1: a sulfate-rich mixture includes an excess of sulfate ions, with respect to the removal of equimolar contents of calcium and sulfate (70% of samples). The most important ions of this mixture type (median) derived from the dataset in order of magnitude are SO_4_^2−^, Na^+^, K^+^, NO_3_^−^, Cl^−^, and Mg^2+^.
- Type 2: calcium-rich mixture includes an excess of calcium ions, with respect to the removal of equimolar contents of calcium and sulfate (30% of samples). The most important ions of this mixture type (median) derived from the dataset in order of magnitude are NO_3_^−^, Ca^2+^, Cl^−^, Na^+^, K^+^, and Mg^2+^.
- Type 3: a mixture containing an important content of carbonates related to an excess of sodium or potassium (relevant within 3% of samples in Type 1).

The results presented focusses on mixtures Types 1 and 2. The data were used as direct input for the ECOS/RUNSALT model^27,36^. The model has equivalent principles to the molality based model, generally known as the Pitzer–Simonson–Clegg model, includes ion concentrations expressed as mole fractions^37^. The outputs of the model are investigated to determine the crystallization behavior of salt mixtures under changing RH between 15 and 95% with a 0.2% resolution at 20 °C. Further input details, limitations, issues, and solutions for the model are taken into consideration, as described in^38^. Special attention is given to certain issues with the representation of hydrate behavior in the outputs, such as calcium nitrates, magnesium sulfates and potassium sulfates. The removal of equimolar contents of calcium and sulfate (CaSO_4_, here regard as the gypsum content (CaSO_4_∙2H_2_O)) is carried out before inputting the ion data into the user interface (RUNSALT) to avoid the software prompting to remove gypsum from the system before it can run the model. Thus, calcium sulfate double salts are not considered in this study, e.g., glauberite (Na_2_Ca(SO_4_)_2_), wattevillite (Na_2_Ca(SO_4_)_2_∙4H_2_O), eugsterite (Na_4_Ca(SO_4_)_3_∙2H_2_O), hydroglauberite (Na_10_Ca_3_(SO_4_)_8_∙6H_2_O), syngenite (K_2_Ca(SO_4_)_2_∙H_2_O), görgeyite (K_2_Ca_5_(SO_4_)_6_∙H_2_O), and polyhalite (K_2_Mg_2_Ca_2_(SO_4_)_4_∙2H_2_O). Carbonate salts and more complex salts such as humberstonite (Na_7_K_3_Mg_2_(SO_4_)_6_(NO_3_)_2_∙6H_2_O), kainite (KMg(SO_4_)Cl∙3H_2_O), Ca(NO_3_)_2_∙KNO_3_∙3H_2_O^39^ and Ca_2_Cl_2_∙Ca(NO_3_)_2_∙4H_2_O^40^ are also not considered in the model.

To identify common salt mixtures and their behavior, datasets are compiled with ion data as mole fraction to derive mean ion values. These values are then used to generate ECOS/RUNSALT outputs. Mole fraction is selected to normalize the quantity of ions between samples and mean values are chosen to maintain charge balance between anions and cations ^34^. The following datasets are evaluated:

- All 11,412 samples (338 sites) (3340 bricks, 3693 mortars, 799 plasters, 1318 stones, and 2262 unidentified).
- 7946 samples identified as Type 1 (sulfate-rich mixture), subsets: 2317 bricks, 2405 mortars, 483 plasters, 1089 stones and 1652 unidentified.
- 3466 samples identified as Type 2 (calcium-rich mixture), subsets: 1022 bricks, 1286 mortars, 316 plasters, 229 stones, and 619 unidentified.

Additional subsets are considered which focus on a sampling depth from the material surface to a depth of maximum 2 cm, and a total salt content equal or greater than 0.8 wt.% (excl. CaSO_4_) compared to the dry material mass. The depth is selected due to practical considerations of sampling depths on site and because the first centimeters of a substrate contain the highest salt load directly influenced by external environmental conditions. The salt content value selected is derived from on-site investigations where limited damage is seen at lower salt contents. This subset results in a total of 1867 samples from 218 sites:

- 921 samples from 186 sites are allocated to Type 1 (sulfate-rich mixture)
- 946 samples from 132 sites are identified as Type 2 (calcium-rich mixture)

The data of all samples are further investigated to evaluate the distribution of mixture types per site considering different sampling heights classified in groups between 0 to 30 cm (including samples taken at the base of a building near the ground), 30 to 50 cm, 50 to 100 cm, 100 to 200 cm, 200 to 400 cm, and above 400 cm, the latter often reaching ceiling vaults. Hereby, evaluating the occurrence of sulfate- and calcium-rich mixtures to understand salt distribution in terms of fractionation as described by Arnold and Zehnder^25^.

ECOS/RUNSALT outputs of all samples are generated to identify common solids as a percentage within the given mixture Types 1 and 2. For each sample, eight iterations of ECOS are run to achieve a resolution of 0.2% RH, thus for each 10% between 15 and 95% RH (removing duplicates), all calculated at 20 °C. The median, minimum and maximum mutual crystallization and dissolution relative humidity points ($RH_{{{\text{cry}}}}^{{\text{m}}}$ and $RH_{{{\text{dis}}}}^{{\text{m}}}$, respectively) are determined for each solid from all RUNSALT outputs (see^38^ for a detailed explanations of these terms and nomenclature). The RH ranges are compared to the relative humidity equilibrium of the individual solids (single salt behavior) to outline the range of crystallization and dissolution RH points of salts in a mixture.

The composition and behavior of mixtures are analyzed, specifically focusing on crystallization under changing relative humidity (RH) at 20 °C and the mutual deliquescence of common solids. Understanding the RH ranges for crystallization and dissolution in different mixtures can help us understand the damage phenomena in weathered stones due to repeated phase changes. This study also investigates the distribution of ions and changes in mixture composition in traditional building materials, particularly regarding their influence on crystallization behavior under varying RH conditions.

## Results and discussion

**Figure 2:**
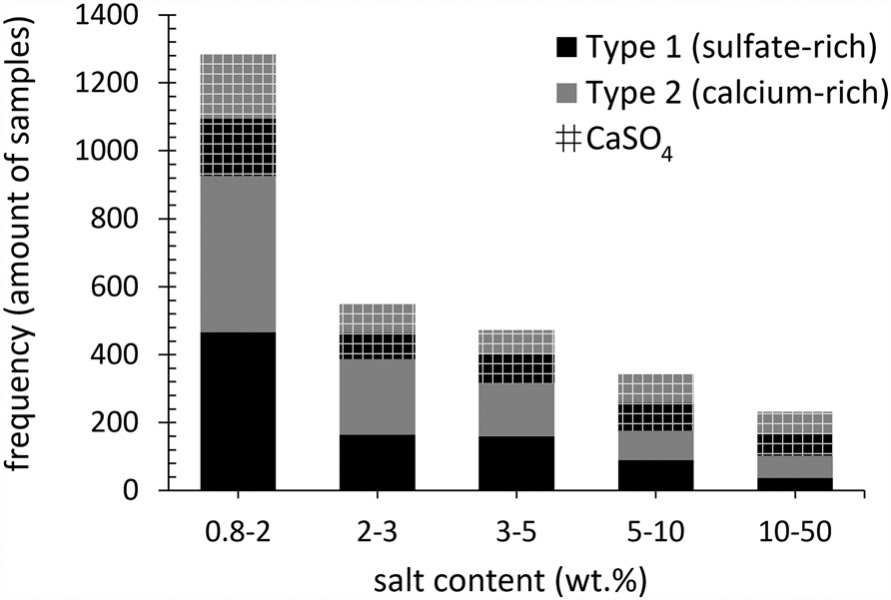
The total salt content (*x*-axis, bin range wt.%–wt.%) per mixture type (excl. CaSO_4_), with Type 1: sulfate-rich (black) and Type 2: calcium-rich (gray), and the CaSO_4_ content per mixture type (grid) as a frequency of the samples (*y*-axis). The dataset is limited to samples with a depth from the material surface to 2 cm and a total balanced ion content ≥ 0.8 wt.% (excl. CaSO_4_). 1867 samples from 218 sites in total, with 921 samples from 186 sites allocated to Type 1 mixtures, and 946 from 132 sites to Type 2. Excluded from the chart are 13 samples with a total salt content above 50 wt.%.

The 338 sites have been investigated for a wide variety of salt-induced deterioration of porous building materials. Besides the salt mixture composition, the salt concentration is indicative for decay, where sufficient pore filling occurs, and daily environmental conditions influence the substrate^3,17^. Thus, the first centimeters of the drying front of the substrate are the primary concern when evaluating the already induced deterioration, while the salt distribution at further depths allows the understanding of salt transport properties over time and possible concerns for treatment methods. The frequency of samples taken from the first two centimeters of the substrates that include a total salt content ≥ 0.8 wt.% (including Cl^−^, NO_3_^−^, Na^+^, K^+^, Mg^2+^, and SO_4_^2−^ or Ca^2^^+^) is shown in Fig. 2, as well as the equimolar content of calcium and sulfate (CaSO_4_), considered as the theoretical gypsum content (CaSO_4_∙2H_2_O), as a function of the two mixture types of interest.

In the first two centimeters of the substrate where salt weathering to stone is mostly visible, approximately 50% of the 1867 samples have a total salt content between 0.8 and 2 wt.% (excl. CaSO_4_). Both mixture types are almost equally represented over the samples, with 466 and 460 allocated to mixture Type 1 (sulfate-rich) and Type 2 (calcium-rich), respectively. The frequency of samples with salt contents above 2 wt.% declines significantly, indicating that a salt content between 0.8 wt.% and 2 wt.% is more common. Gypsum is always present and remains relatively similar in terms of frequency from 0.8 wt.% upwards. However, what is most important to note is the almost equal distribution of the two mixture types within this subset, with 921 samples from 186 sites allocated to Type 1 mixtures, and 947 from 132 sites to Type 2 mixtures. While in the complete dataset (11,412 samples), thus including all sampling depths (as detailed in^33^) a higher frequency of up to two thirds was observed for Type 1 mixtures, indicating that sulfate-rich mixtures tend to be further distributed in depth.

The analysis of the complete dataset (11,412 samples) reveals the frequency of different ions, with at least five charge-balanced ions included in > 92% of all samples, indicating the significance of mixtures opposed to single salts. The only ion that occurs less frequently is Mg^2+^, which is present in 66% of the samples. When focusing on the two mixture types, Mg^2+^ occurs in 86% of all Type 1 and in 56% of all Type 2 samples. Additionally, NO_3_^−^ occurs slightly less in all Type 1 samples at 88%. The ion distribution within the complete dataset is further detailed in^33^ and the most important ion mixtures are presented in Table 1.

**Table 1:** Representation of ions in common mixture compositions considering two mixture types. Left Type 1 (sulfate-rich) mixture and right Type 2 (calcium-rich) mixture, followed by the complete mixture of seven ions including calcium and sulfate (considered as gypsum). > 92% of 11,412 samples from the built environment contain at least five ions, thus four mixture compositions (T1_V_, T1_VI_, T2_V_, T2_VI_) are investigated further with ECOS/RUNSALT with mean ion values derived from the dataset.

	Type 1											Type 2											
								Na^+^		SO_4_^2−^		Ca^2+^		NO_3_^−^									
						K^+^		Na^+^		SO_4_^2−^		Ca^2+^		NO_3_^−^		Cl^−^							
				NO_3_^−^		K^+^		Na^+^		SO_4_^2−^		Ca^2+^		NO_3_^−^		Cl^−^		Na^+^					
T1_V_		Cl^−^		NO_3_^−^		K^+^		Na^+^		SO_4_^2−^		Ca^2+^		NO_3_^−^		Cl^−^		Na^+^		K^+^			T2_V_
T1_VI_	Mg^2+^	Cl^−^		NO_3_^−^		K^+^		Na^+^		SO_4_^2−^		Ca^2+^		NO_3_^−^		Cl^−^		Na^+^		K^+^		Mg^2+^	T2_VI_
					K^+^		Na^+^		SO_4_^2−^		Ca^2+^		NO_3_^−^		Cl^−^		Mg^2+^						

A separation of salt mixture types, as described by Arnold and Zehnder^25^, with more Type 1 (sulfate-rich mixture, including less soluble solids, thus less hygroscopic) nearer to the ground, and more Type 2 (calcium-rich mixture, including more soluble solids, thus more hygroscopic) at greater heights is likely. However, no statistical significance was obtained from the full dataset as a 0.95 *p*-value was determined from a chi-square test between the number of samples per mixture type identified at different heights: 0 to 30, 30 to 50, 50 to 100, 100 to 200, 200 to 400, and greater than 400 cm. This indicates that both mixture types are possible at any given height. However, both are most often found between a height of 100 and 200 cm. This height primarily experiences salt accumulation and decay, particularly when ground water is, or was, readily available to migrate upwards due to the capillary forces within the porous media.

**Figure 3:**
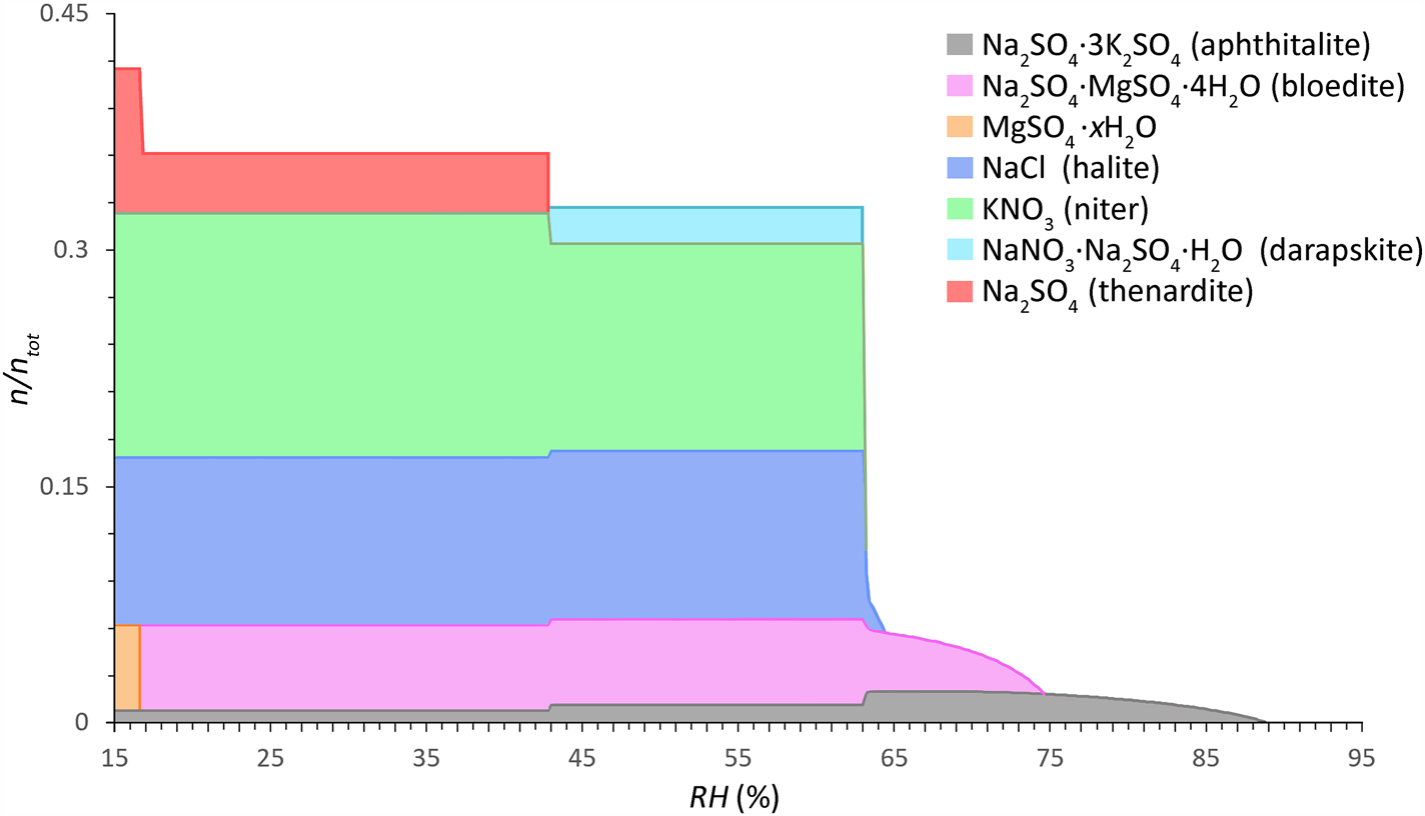
Common salt mixture behavior of Type 1 mixtures (sulfate-rich, less hygroscopic) calculated between 15 and 95% RH (0.2% resolution) (*x*-axis) at 20 °C. The relative amount of substance is given as a fraction of crystalline salt ($n/n_{{{\text{tot}}}}$) (*y*-axis), mean of 921 samples from 186 sites (considering a maximum sampling depth from the surface to 2 cm and a total salt content ≥ 0.8 wt.% (excl. CaSO_4_)). Mole fractions: Cl^−^: 0.1070, NO_3_^−^: 0.1543, SO_4_^2−^: 0.1771, Na^+^: 0.3063, K^+^: 0.2012, and Mg^2+^: 0.0540. Modified ECOS/RUNSALT output^27,36^.

**Figure 4:**
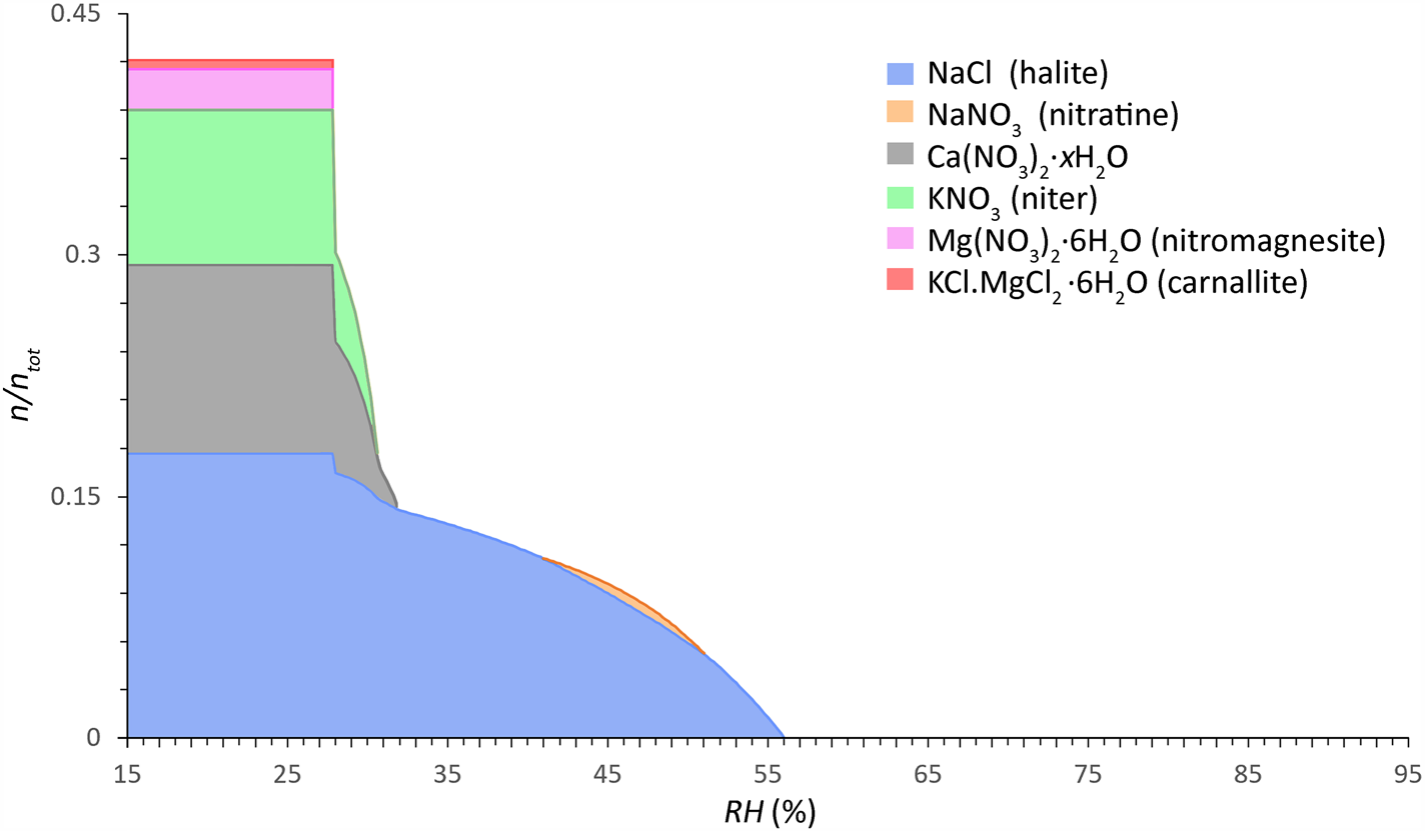
Common salt mixture behavior of Type 2 mixtures (calcium-rich, more hygroscopic) calculated between 15 and 95% RH (0.2% resolution) (x-axis) at 20 °C. The relative amount of substance is given as a faction of crystalline salt ($n/n_{{{\text{tot}}}}$) (y-axis), mean of 946 samples from 132 sites (considering a maximum sampling depth from the surface to 2 cm and a total salt content ≥ 0.8 wt.% (excl. CaSO_4_)). Mole fractions: Cl^−^: 0.1937, NO_3_^−^: 0.3801, Na^+^ : 0.1770, K^+^ : 0.1016, Mg^2+^ : 0.0308 and Ca^2+^ : 0.1168. Modified ECOS/RUNSALT output^27,36^.

Moving forward to the identification of common mixture types, mean values of the ion content for the different subsets and groups are used as input data for the ECOS/RUNSALT model to evaluate the crystallization behavior of the mixture compositions. The outputs show common mixtures for each dataset as specified in the method section. Similar mixture composition and behavior under changing RH conditions are identified, with minor differences in the number of solids and RH ranges of interest per mixture type. Furthermore, no significant differences are seen in the solids shown in the plots between the different heights, depths, and materials (brick, mortar, plaster, and stone). The mean ion values of the two mixture types derived from all samples (all materials and heights) taken from the material surface to a depth of maximum 2 cm and a total salt content ≥ 0.8 wt.% (excluding CaSO_4_) are shown in Figs. 3 and 4. The results present common mixture types and solids, and their behavior under changing relative humidity.

Common solids identified in Type 1 mixtures are: aphthitalite (Na_2_SO_4_∙3K_2_SO_4_), bloedite (Na_2_SO_4_∙MgSO_4_∙4H_2_O), magnesium sulfates (MgSO_4_∙*x*H_2_O), halite (NaCl), niter (KNO_3_), darapskite (NaNO_3_∙Na_2_SO_4_∙H_2_O), and thenardite (Na_2_SO_4_). Type 2, on the other hand, includes halite, niter, nitratine (NaNO_3_), calcium nitrates (Ca(NO_3_)_2_*∙x*H_2_O), nitromagnesite (Mg(NO_3_)_2_∙6H_2_O), and carnallite (KCl∙MgCl_2_∙6H_2_O). Three salts occur in both mixture types: halite, niter, and nitratine, the latter however is part of the double salt darapskite in Type 1. Solids that are shown in the plots that include magnesium can simply be removed without significant changes to the behavior when considering five ion mixtures (Cl^−^, NO_3_^−^, Na^+^, K^+^, and SO_4_^2−^ or Ca^2+^). The outputs are evaluated following the method described in^38^, with an example output provided and a summary of the reactions under drying conditions for a common Type 1 mixture with five ions. From the common mixture compositions and behaviors presented in Figs. 3 and 4 the statements made by Steiger et al.^14^ and Arnold and Zehnder^25^ are verified in the presented analysis based on the relative composition of the two mixture types, with:

- **Type 1:** a sulfate-rich mixture is less hygroscopic: SO_4_^2−^, Na^+^, K^+^, NO_3_^−^, Cl^−^, Mg^2+^
- **Type 2:** a calcium-rich mixture is more hygroscopic: NO_3_^−^, Ca^2+^, Cl^−^, Na^+^, K^+^, Mg^2+^

Specific trends are derived from the outputs for both mixture types. Here, the common solids and mutual crystallization RH (with a resolution of 0.2%) at 20 °C for the Type 1 mixture under drying conditions are (cf. Fig. 3): 88.8% for aphthitalite, 74.6% for bloedite, 64.4% for halite, 63.2% for niter, and 63% for darapskite. Partial decomposition of aphthitalite occurs under drying conditions below 65% and 43%, during the first step this coincides with an increase of bloedite and crystallization of halite, niter and darapskite, followed by a slight increase of niter and thenardite, respectively. The latter is a solid-state reaction and overlaps with the decomposition of darapskite. The last solid-state reaction occurs at 17% when bloedite decomposes completely to form MgSO_4_∙*x*H_2_O and thenardite. Further research is needed to verify the solid-state reactions and identify the magnesium sulfate hydrates, as detailed in ^38^.

For the Type 2 mixture, the common solids and mutual crystallization RH of interest are (cf. Fig. 4): 56% for halite, 51% for nitratine, 31.8% for Ca(NO_3_)_2_∙*x*H_2_O, 30.6% for niter, and 27.8% for both nitromagnesite and carnallite. The solid phase Ca(NO_3_)_2_ was incorrectly calculated by ECOS as the anhydrous phase is not able to form under these climatic conditions. The only feasible form of crystallization is the tetrahydrate (nitrocalcite), however its crystallization is often observed to be kinetically hindered. Likewise, an unverified phenomenon is shown with nitratine as it decomposes below 41%, returning to solution as the amount of crystalline NaCl and the solution concentration increases.

**Figure 5:**
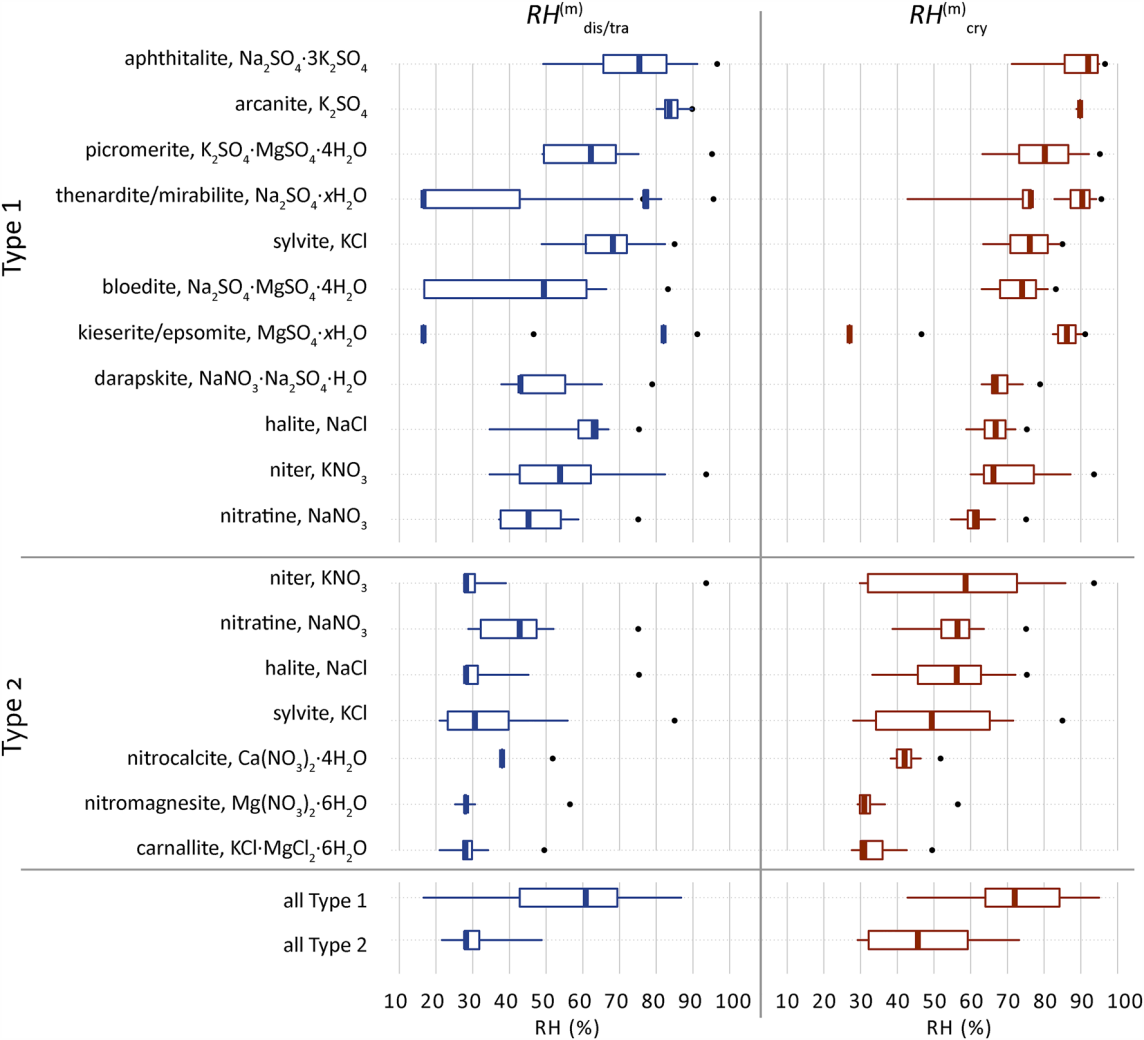
Distribution of the mutual crystallization and dissolution or transition relative humidity points ($RH_{{{\text{cry}}}}^{{\text{m}}} ,RH_{{{\text{dis}}}}^{{\text{m}}} ,RH_{{{\text{tra}}}}^{{\text{m}}}$) of common solids identified for Type 1 (sulfate-rich) or Type 2 (calcium-rich) mixtures, limited to solids that occur in at least 20% of samples per mixture type, here excluding three solids per mixture type in addition to gypsum. Solids are identified from the ECOS outputs of all 11,412 samples. Boxplots: 25% left, 75% right, with whiskers drawn to the 5th and 95th percentiles, excluding outliers. The dots indicate the single salt $RH_{{{\text{cry}}}}^{{}} ,RH_{{{\text{dis}}}}^{{}} ,RH_{{{\text{tra}}}}^{{}}$. Solids with a variety of possible hydrate phases are shown as *x*H_2_O and their calculated RH points of interest are shown with increasing hydrate from left to right, excluding unverified metastable phases. The crystallization behavior has been calculated between 15 and 95% at 20 °C with a resolution of 0.2%. Corrections are applied following identified issues for MgSO_4_, Ca(NO_3_)_2_, and K_2_SO_4_^38^. The two last boxplots (all Type 1 and 2) show a summary of all solids identified including ones below 20% of samples per mixture type.

Common solids and their behavior are derived from the ECOS outputs of all 11,412 samples and presented in Fig. 5. The RH-range (distribution) of the solid behavior in the mixture compositions are shown and grouped per mixture type. The median, minimum and maximum mutual crystallization, and dissolution/transition relative humidity points ($RH_{{{\text{cry}}}}^{{\text{m}}} ,RH_{{{\text{dis}}}}^{{\text{m}}} ,RH_{{{\text{tra}}}}^{{\text{m}}}$) are determined for each solid from all samples and compared to the single salt $RH_{{{\text{cry}}}}^{{}} ,RH_{{{\text{dis}}}}^{{}} ,RH_{{{\text{tra}}}}^{{}}$. See^38^ for a detailed explanations of these terms and nomenclature.

Additionally, the distribution of the behavior of all solids per mixture type including less frequent ones (occurring in less than 20% of samples per mixture type) are summarized to evaluate the overall possible behavior of the type. The *RH* points for the (single) salt are the same in both sides of Fig. 5, to clarify the $RH_{{{\text{cry}}}}^{{}}$ and $RH_{{{\text{dis}}}}^{{}}$ of the salt is identical to the RH equilibrium ($RH_{{{\text{eq}}}}^{{}}$). While for solids in a mixture both points can be different, for example as shown in Fig. 4, the $RH_{{{\text{cry}}_{{{\text{hal}}}} }}^{{\text{m}}}$ for halite is 56% and the $RH_{{{\text{dis}}_{{{\text{hal}}}} }}^{{\text{m}}}$ is 27.8%.

From Fig. 5, common solids are identified that occur in at least 20% of the samples per mixture type: eleven in Type 1 and seven in Type 2. The RH points of interest are vastly different compared to the relative humidity equilibrium for the corresponding individual salts, with the critical crystallization RH of salts in a mixture always below the $RH_{{{\text{eq}}}}^{{}}$ of the (single) salt. A main RH threshold at 60% is identified for the median mutual crystallization RH at 20 °C for all common solids. The solids identified in Type 1, excluding kieserite, show a median $RH_{{{\text{cry}}}}^{{\text{m}}}$ above 60% RH, and for solids in Type 2 they remain below this value.

The median trends of all solids between the $RH_{{{\text{dis}}}}^{{\text{m}}}$ and $RH_{{{\text{cry}}}}^{{\text{m}}}$ are 61% and 72% for Type 1, and 28% and 46% for Type 2, respectively. These median values for both mixture types are the critical RH ranges in which common mixtures will dissolve and crystallize, thus causing decay. However, the median, maximum and minimum RH ranges between the $RH_{{{\text{dis}}}}^{{\text{m}}}$ and $RH_{{{\text{cry}}}}^{{\text{m}}}$ are also defined for each solid in all the mixture compositions and indicate the range in which the solid can crystallize and dissolve. It is important to consider this for each individual solid in the given mixture separately, while keeping in mind that the phase transitions shown for sodium and magnesium sulfate can either exist as the specific phase given or a phase transition can occur in the mixture. The latter was not evaluated in terms of frequency, thus keeping in mind that phase transitions ((de)hydration) of a single solid can or cannot occur in a given mixture, and that the single salts related to a double salt often coexist or form within the given crystallization pathway.

The results of the statistical analysis show 14 common salts that occur in at least 20% of samples per mixture type. The frequency of occurring solids is further defined in Table 2. Five of these solids are double salts, aphthitalite, bloedite, darapskite, and picromerite, found in Type 1 and carnallite is frequently identified in Type 2 mixtures. Less frequent solids that are not included in the table are for Type 1: nitromagnesite (Mg(NO_3_)_2_∙6H_2_O) (10.2%), carnallite (KCl∙MgCl_2_∙6H_2_O) (2.6%), and bischofite (MgCl_2_∙6H_2_O) (0.6%), and for Type 2: antarcticite (CaCl_2_∙6H_2_O) (15.8%), bischofite (MgCl_2_∙6H_2_O) (3.5%), and MgCa(NO_3_)_4_∙10H_2_O (2.9%). Remarking that the results presented do not include the amount of solid as they appear in the mixtures.

**Table 2:** Common solids and their frequency of occurrence (%) as identified in the samples per mixture type, excluding solids below a frequency of 20% and anhydrite/gypsum. (B) indicates solids that occur in both mixture types. (E) shows salts that overlap with the ones identified in the ECOS outputs of the mean ion values per mixture type. *x*H_2_O implies different possible states of hydration, while (m) indicates unverified metastable phases not considered.

	Common solids	Frequency %
Type 1 Sulfate-rich mixture	(B) (E) niter, KNO_3_	86%
	(B) (E) halite, NaCl	86%
	(E) aphthitalite, Na_2_SO_4_∙3K_2_SO_4_	72%
	(E) thenardite/(m)/mirabilite, Na_2_SO_4_∙*x*H_2_O	69%
	(E) kieserite/(m)/epsomite, MgSO_4_∙*x*H_2_O	56%
	(E) bloedite, Na_2_SO_4_∙MgSO_4_∙4H_2_O	48%
	(E) darapskite, NaNO_3_∙Na_2_SO_4_∙H_2_O	46%
	(B) nitratine, NaNO_3_	30%
	arcanite, K_2_SO_4_	24%
	(B) sylvite, KCl	21%
	picromerite, K_2_SO_4_∙MgSO_4_∙6H_2_O	20%
Type 2 Calcium-rich mixture	(B) (E) halite, NaCl	100%
	(E) (m)/nitrocalcite, Ca(NO_3_)_2_∙*x*H_2_O	100%
	(B) (E) niter, KNO_3_	79%
	(E) nitromagnesite, Mg(NO_3_)_2_∙6H_2_O	65%
	(B) (E) nitratine, NaNO_3_	60%
	(E) carnallite, KCl∙MgCl_2_∙6H_2_O	47%
	(B) sylvite, KCl	23%

Four solids, halite (NaCl), niter (KNO_3_), nitratine (NaNO_3_), and sylvite (KCl) occur in both mixture types. Above a frequency of 45% the solids overlap identically with the results of the RUNSALT outputs of the mean ion values considering a maximum sampling depth from the surface to 2 cm, as shown in Figs. 3 and 4. Thus, concluding with the identification of the most common solids found in the built environment with seven solids identified in Type 1 and six in Type 2 mixtures.

It remains important to note that due to kinetics and possible separation of solids from the solution during crystallization in realistic situations, that is, the in-pore situation, deviations from the modeled crystallization pathway can take place. Additionally, the individual salts of each double salt can occur, resulting in an increase of the frequency regarding the solids arcanite (K_2_SO_4_), sodium sulfates (Na_2_SO_4_∙*x*H_2_O), magnesium sulfates (MgSO_4_∙*x*H_2_O), and nitratine (NaNO_3_) in Type 1, and in Type 2 mixtures sylvite (KCl) and bischofite (MgCl_2_∙6H_2_O). Furthermore, the result corresponds well with efflorescence detected on sites, the frequency was compared to reported efflorescence in 112 journal articles and conference papers^41^. Excluding calcium sulfate and related double salts, the salts as efflorescence relevant to this study are presented as a percentage of times they were mentioned in these papers: halite (49%), thenardite (45%), niter (35%), nitronatrite (32%), epsomite (30%), aphthitalite (23%), mirabilite (22%), hexahydrite (21%), syngenite (20%), darapskite (13%), nitrocalcite (11%), arcanite (7%), starkeyite (7%), picromerite (7%), bloedite (5%), and 19 others below 5%, of which kieserite, antarcticite, and pentahydrate (MgSO_4_∙5H_2_O). Naturally, more hygroscopic salts are less likely to effloresce on the surface of in-situ building materials.

In future research, it would be beneficial to examine the different mixture types and their connections to decay phenomena observed on-site. Type 1 mixtures include more hydrating and double salts and would cause, for example, visible efflorescence and sub/crypto-florescence with severe powdering, material disintegration and/or delamination under regular RH fluctuations around 61% and 72%. While Type 2 mixtures are more likely to result in moisture stains, biological contamination and/or surface powdering caused by RH fluctuations around 28% and 46%. These statements can be verified through on-site investigations and experimental determination of salt mixture kinetics over time, including solid state reactions.

The kinetic aspects require specific attention toward a better understanding of crystallization cycles under realistic climatic conditions within the pore structure of the material. Hereby focusing on pore filling, crystallization pressure, changes in the solution viscosity, separation of solids from the solution, pore clogging effects, capillarity, crystallization pathways and climatic buffering of the material. Moreover, the study did not delve into the quantification of the solids identified, which would enhance our comprehension of the extent of these solids in typical mixture composition. Finally, certain hydrating salts and double salt phases need further attention, such as magnesium sulfates, calcium nitrates, potassium calcium nitrates, humberstonite, and calcium sulfate double salts. When evaluating the risk of material decay from salt mixtures and understanding the damage mechanisms over time under changing climate conditions, it is crucial to take all these factors into account. This information can then be used to develop preventive measures, evaluate the potential for decay, and guide the design of future experiments.

## Conclusions

Common salt mixtures in the built environment, governed by the behavior of two mixture types, were investigated. Upon removal of equimolar amounts of calcium and sulfate, Type 1 mixtures (sulfate-rich) and Type 2 mixtures (calcium-rich) were identified. This process is based on gypsum crystallizing rapidly from a solution because of its low solubility. Due to the formation of more hydrating and sulfate-containing (double) salts in Type 1 these tend to be less hygroscopic, while the presence of more nitrate salts in Type 2 cause these to be more hygroscopic by nature. The data showed that in 92% of samples at least five ions are available, with magnesium seen less frequently. Thus, four common mixtures are identified including sodium, potassium, nitrate, chloride, and calcium or sulfate, either including or excluding magnesium.

The mean ion data entered in the thermodynamic model (ECOS/RUNSALT) showed common solids and their behavior for each mixture type. In Type 1 these are niter, halite, aphthitalite, sodium and magnesium sulfate (phases), bloedite, and darapskite, while for Type 2 these are halite, calcium nitrate, niter, nitromagnesite, nitratine, and carnallite. The same common solids were identified, occurring with a frequency in at least 46% per mixture type, from the statistical analysis of all 11,412 modeled samples. Salts that occur in at least 20% of the samples per mixture type showed 14 common solids, which is a valuable reduction of the more than 85 possible phases to consider. The modeled crystallization behavior of all mixtures revealed an overview of the vast range of possible mutual crystallization and dissolution RH ranges of the 14 common solids compared to the (single) salt behavior. These results are important to understand the overall behavior of salts in terms of RH ranges in which they can crystallize and dissolve in real world situations, where a mixture of ions is always present.

If building materials display signs of salt damage or moisture stains, even in the absence of liquid water, it has been commonly observed that the total salt content (excluding gypsum) in the first two centimeters is within the range of 0.8–2 weight percent relative to the dry material weight. Both mixture types occur with a similar frequency in the first two centimeters of the substrate in different materials and at different heights. The main distinction is that Type 1 mixtures are more commonly found at greater depths, while Type 2 mixtures tend to accumulate closer to the surface. Both mixture types are present together in 64% of the 338 sites, indicating the importance of sampling different areas of one site. It is important to consider the presence of liquid water, as gypsum is often found in high quantities in nearly all samples and is likely the primary source of damage in such cases. While, when liquid water is not or no longer present a total salt content (excluding gypsum) of approximately 1 wt.% is sufficient to cause damage or moisture related problems in a wide range of porous materials, dependent on the past and future climatic conditions. This value is determined by the material properties and any external factors that may impact the cycles of salt crystallization over time.

## Data Availability

Data are available for this paper on request. Correspondence and requests for materials should be addressed to S.G.

## Code availability

Codes are available for this paper on request. Correspondence and requests for materials should be addressed to S.G. or S.A.O.
